# Chinese Medicine Formula Lingguizhugan Decoction Improves Beta-Oxidation and Metabolism of Fatty Acid in High-Fat-Diet-Induced Rat Model of Fatty Liver Disease

**DOI:** 10.1155/2013/429738

**Published:** 2013-05-02

**Authors:** Tao Liu, Li-Li Yang, Lu Zou, Dong-Fei Li, Hong-Zhu Wen, Pei-Yong Zheng, Lian-Jun Xing, Hai-Yan Song, Xu-Dong Tang, Guang Ji

**Affiliations:** ^1^Institute of Digestive Diseases, Longhua Hospital, Shanghai University of Traditional Chinese Medicine, Shanghai 200032, China; ^2^Shanghai East Hospital, Tongji University, Shanghai 200120, China; ^3^Xiyuan Hospital of China Academy of Chinese Medical Sciences, Beijing 100091, China; ^4^E-Institute of Shanghai Municipal Education Commission, Shanghai University of Traditional Chinese Medicine, Shanghai 201203, China

## Abstract

Lingguizhugan decoction (LGZG), a classic traditional Chinese medicine (TCM) formula, has been used to treat obesity and hyperlipidemia in recent years, but the related mechanisms underlying the regulation of lipid metabolism by LGZG are not clear yet. Here, we reported the effectiveness and possible mechanisms of LGZG on rats with fatty liver disease induced by high-fat diet (HFD). Our results demonstrated that LGZG significantly attenuated HFD-induced fatty liver disease, as measured by body weight, liver index, epididymal fat pad-body weight ratio (EFP/BW), liver injury, and hepatic triglycerides (TG) probably through increasing serum thyroid hormone levels, improving beta-oxidation (via modulation of TR**β**1 and CPT1A expression), metabolism and transport (through modulation of SREBP-1c, ACSL and ApoB100 expression) of fatty acid. In addition, we discovered the herbal combination with the properties of warming yang to relieve water retention in the formula and proposed the biological basis of LGZG conventional effect via further study on disassembled formula. This study, for the first time, revealed the mechanisms through which LGZG regulates lipid metabolism. Furthermore, our study suggested that it might be feasible to understand the scientific implications of TCM from the perspective of classic formulas' conventional efficacy.

## 1. Introduction

Lingguizhugan (LGZG) decoction is an ancient Chinese herbal formula from a classic clinical book of traditional chinese Medicine (TCM) titled *Jingui Yaolue* for the treatment of diseases caused by phlegm-fluid retention. It is recorded that conditions with phlegm and fluid retention should be modulated by drugs with warm nature, and the specific formula is Lingguizhugan decoction. LGZG is a typical formula established under the therapeutic principle of warming yang to relieve water retention, indicated for phlegm and fluid retention. In the past decade, LGZG has also been applied to diseases with the feature of phlegm and fluid retention, such as chronic congestive heart failure (CHF). A metaanalysis including 280 patients concluded that LGZG increased clinical benefits of inotropes, diuretics and vasodilators therapy in CHF [1]. Furthermore, in animal studies, LGZG was found to improve cardiac function and cardiac endocrine function in rabbit models of CHF [2], reduce serum Ang II, ET-1, TNF-*α*, and IL-1*β* levels, retard adverse ventricular remodeling, and reduce overexpression of cytokines in rat models of CHF [3]. Moreover, LGZG was found to restrain the weight gain caused by antipsychotic medications without any obvious side effect [4], suggesting that LGZG may bring benefits for metabolic syndrome (MS), which is regarded as having the properties of phlegm and fluid retention in TCM. LGZG also demonstrated clinical benefits for hemorheology in hyperlipidemic rats, indicating that it might also be an alternative choice for hyperlipidemia treatment [5, 6]. Taken together, it is of great significance to explore the effect and possible mechanisms of LGZG on nonalcoholic fatty liver disease (NAFLD), the hepatic component of MS with a high prevalence.

Our research team has been working in the field of NAFL treatment with TCM for more than two decades. Our epidemiological evidence [7] indicated that the crucial TCM pathogenesis of NAFL was phlegm and fluid retention, which was primarily caused by spleen-yang deficiency. Furthermore, we found that Warming Yang to Relieve Water Retention eliminated hepatic fat accumulation in NAFL patients [8]. Our previous study has compared the effectiveness of three different classic formulae on hepatic steatosis (as measured by hepatic triglycerides) in HFD-induced rat models of NAFL. These formulae were rooted in three different therapeutic principles, that was, tonifying Qi for spleen invigoration, warming yang for qi activation, and warming yang to relieve water retention. Finally the results demonstrated that LGZG effectively reduced hepatic triglycerides (TG) [9]. Therefore, investigating the mechanisms underlying the effect of LGZG on NAFL will provide another treatment option for NAFL and help us understand the scientific connotation of TCM therapeutic principle Warming Yang to Relieve Water Retention in NAFL treatment.

## 2. Materials and Methods

### 2.1. Drug Preparation and Diet

LGZG comprises four Chinese herbs: *Poria* (20 g), *Ramulus Cinnamomi *(15 g), *Rhizoma Atractylodis Macrocephalae* (15 g), and *Radix Glycyrrhizae* (10 g). The dosage is determined according to the text book of *The Hndouts of Jingui Yaolue *[10]. All herbs were purchased from Longhua Hospital affiliated to Shanghai University of TCM. Herbal decoction was prepared in accordance with conventional TCM decocting methods, briefly, (1) place all herbs in a cooking pot (porcelain) with 500 mL water; (2) boil the herbs with highest heat after 30 minutes of soak; (3) reduce heat and simmer for 20 minutes; (4) transfer the liquid by filtration; (5) add water and boil the remaining, and then repeat (3) and (4) one more time to make a second dose of medicine; (6) mix the two doses in a glass pot. The final concentrated decoction is 100 mL (pure solution). Quality was control under high-performance liquid chromatography (HPLC) as previously described [11], and details were shown in Supplement 1 (see Supplementary Material available online at http://dx.doi.org/10.1155/2013/429738). LGZG was administered at a dose of 10 mL/kg/d (pure solution), which was approximately 7 times of the standard dose in practice, according to the dose-equivalence equation between rats and humans [12]. Meanwhile, the following formulas were prepared based on the herbal combination rule: Lingguizhugan Decoction without *Ramulus Cinnamomi* (LZG, *Poria*, *Rhizoma Atractylodis Macrocephalae*, *Radix Glycyrrhizae*), Lingguizhugan Decoction without *Poria* (GZG, *Ramulus Cinnamomi*, *Atractylodis Macrocephalae*, *Radix Glycyrrhizae*), Lingguizhugan Decoction without *Rhizoma Atractylodis Macrocephalae* and *Radix Glycyrrhizae* (LG, *Poria*, *Ramulus Cinnamomi*). Methods used in decoction and quality control were the same as that used in LGZG, and equal doses were administered. Ordinary diet and HFD (consists of 10% lard oil, 2% cholesterol, and 88% STD) were obtained from Shanghai Si-Lai-Ke Experimental Animal Ltd. (Shanghai, China).

### 2.2. Animals and Interventions

SPF animals (male Wistar rats, 130 g ± 10 g) were obtained from Shanghai Si-Lai-Ke Experimental Animal Ltd. (Shanghai, China). Rats were housed in an SPF, temperature- (24°C ± 2°C) and humidity-controlled (55% ± 10%) room with a 12-hour light-dark cycle (commencing with light at 08:00) in the experiment center, Longhua Hospital affiliated to Shanghai University of TCM. Animals were randomized into 6 groups: normal group, model group, LGZG group, LZG group, GZG group, and LG group (*n* = 8).

Studies began after an acclimation period of 1 week. Rats in the normal group were fed with ordinary diet, while the rats in the other groups were fed with HFD. Food and drinking water/herbal decoction were supplied ad libitum. Treatment lasted 5 weeks, and drug concentration was titrated every other day according to the daily liquid intake of animals. 

Five weeks later, the animals were sacrificed under pentobarbital sodium (2%, 5.5 mL/kg) anesthesia following a 12-hour fast. Blood samples were collected in serum tubes from the abdominal aorta. Two samples (1.0 cm × 1.0 cm × 0.2 cm) from the identical lobe and position in the liver were obtained and then fixed in 10% neutral-buffered formalin. The remaining liver was stored at −80°C until use.

All animal procedures were approved by the Animal Experiment Ethics Committee of Shanghai University of TCM.

### 2.3. Pathology

Liver samples were fixed in 10% formalin for 48 hours and then routinely processed to paraffin. Sections which cut on a microtome at a thickness of 4 *μ*m were stained with hematoxylin and eosin (H & E), and then microscopic images were obtained.

### 2.4. Biochemical Analysis

Almandine aminotransferase (ALT), aspartate aminotransferase (AST), triglyceride (TG), total cholesterol (TC), high-density lipoprotein cholesterol (HDL-C), and low-density lipoprotein Cholesterol (LDL-C) were measured with auto analyzer (HITACHI 7170, Japan). 

TG concentration was assayed using kits obtained from Nanjing Jiancheng Bioengineering Institute following the manufacturers' instructions. 200 mg liver tissues were obtained and snipped into small pieces. These tissues were placed in 3 mL of ethanol-acetone mixture (v : v = 1 : 1), homogenized on ice at 10000–20000 rpm/min for 20 seconds (repeat 2-3 times), and then stored at 4°C overnight. In the next day, samples were centrifuged at 3000 rpm/min for 20 minutes at 4°C to obtain the supernatant. 

3,5,3-Triiodothyronine (T3), free triiodothyronine (FT3), total thyroxine (T4), and free thyroxine (FT4) were measured with Roche Diagnostics GmbH (E170, Roche Diagnostics GmbH, Mannheim, Germany). 

### 2.5. Real-Time PCR for mRNA Analysis

TR*β*1, CPT1A, SREBP-1c, ApoB100, and ACSL mRNA levels were determined by real-time PCR. Primers were designed using the Primer premier 5.0 software (Table 1). 

Quantitative measurement was performed using the Premix Ex Taq kit (TakaRa) according to the manufacturer's instructions on Applied Biosystems StepOne Plus Sequence Detection System. The real-time cycler conditions were as follows: first denaturated at 95°C for 30 sec and then amplified with 40 cycles (each cycle was denaturation at 95°C for 5 s and annealing/extension at 60°C for 30 min). Product purity was confirmed by dissociation curve analysis. Gene expression was quantified relative to the values of the control group after adjusting for *β*-actin by the 2^−ΔΔCT^ method as described previously [13].

### 2.6. Protein Isolation and Western Blotting

Three liver samples of each group were homogenized in liquid nitrogen, and whole-cell protein was extracted by using lysate buffer containing proteinase inhibitor (complete ULTRA Tablets, minipore). Protein concentration was quantified spectrophotometrically (Bioiek H4) by using BSA protein assay kit (thermo). Protein samples were separated by PAGE using 10% SDS-polyacrylamide gels. Samples were transferred to polyvinylidene fluoride membrane (Immobilon-P transfer membrane, Millipore) and blocked with 5% milk. The membrane was incubated with a mouse anti-TR*β*1 primary antibody (1 : 100, Santa Cruz) for 1.5 h at room temperature followed by the secondary antibody (against mouse, Cell Signaling) for 1 h at room temperature. The primary antibodies including rabbit anti-CPT1A (1 : 500, proteintech), and mouse anti-SREBP-1 (1 : 250, abcam), rabbit anti-ACSL (1 : 500, proteintech), mouse anti-beta-actin (1 : 1000, abcam) were similar. Lastly, each protein band was detected using enhanced chemiluminescence (ECL, Millipore). The densitometric values were measured with Gel-Pro Analyzer.

### 2.7. Statistical Analysis

Data were expressed as mean ± SD and were analyzed by one-way analysis of variance (ANOVA), LSD-*t*, or Games-Howell as appropriate. The statistical significance was defined as two-sided *P* value of <0.05. Statistical analyses were performed using SPSS 16.0 software (SPSS, Chicago, USA).

## 3. Results

### 3.1. Rats' Body Weight, Liver Index, and Epididymal Fat Pad-Body Weight Ratio (EFP/BW) Were Reduced by LGZG

Body weight, liver index, and EFP/BW in model group were significantly higher than that in normal group (*P* < 0.05). All four formulas decreased liver index and EFP/BW compared to model group, though statistical significance was only detected in LGZG and LG groups (*P* < 0.05). This effect was not significantly different between LZG and GZG groups (*P* > 0.05, Table 2). 

### 3.2. Hepatic Injury and TG Were Improved by LGZG

**Figure 1:**
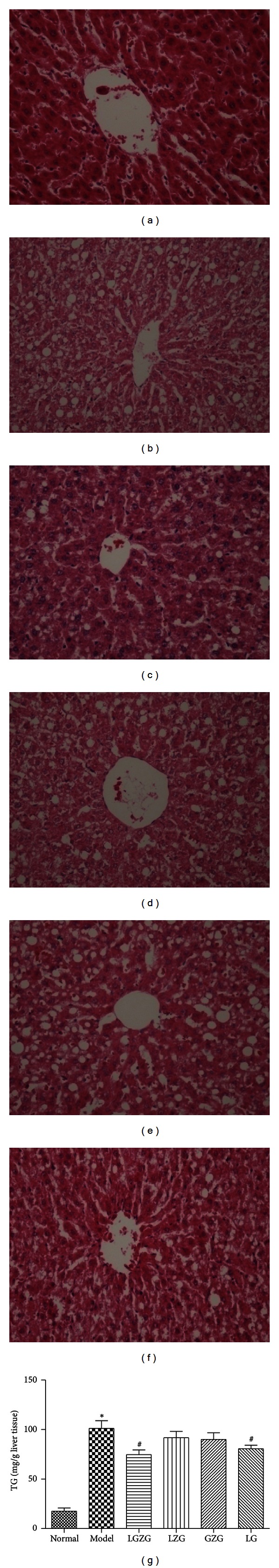
The influence of LGZG and its decomposed recipes on rats' histopathology (H&E stain ×200) and TG levels. (a) Normal group fed with chow diet; (b) model group fed with High-Fat Diet (HFD); (c) LGZG group fed with HFD and LGZG; (d) LZG group fed with HFD and LZG; (e) GZG group fed with HFD and GZG; (f) LG group fed with HFD and LG. (g) The influence of LGZG and its decomposed recipes on rats' hepatic TG. *P < 0.05 versus normal group, ^#^ *P* < 0.05 versus model group.

Histological analysis showed that, in normal group (Figure 1(a)), the central vein (CV) was surrounded by hepatocytes arranged radially in plates, and the blue-stained nucleus was located in the centre of the cell. In model group (Figure 1(b)), hepatocytes were not only disordered with loosed cytoplasm and ballooning degeneration but also abundant marked lipid droplets and vacuolation presented; the blue-stained nucleus was located in the border of the cell. In LZG (Figure 1(d)) and ZGZ groups (Figure 1(e)), they were similar to that in the model group. On the contrary, in LGZG (Figure 1(c)) and LG (Figure 1(f)) groups, the lipid droplets were obviously reduced comparing with the model group, and the hepatocytes arrangement improved. LGZG effect was superior than LG. 

The level of hepatic TG (Figure 1(g)) in model group was significantly higher than that in normal group (*P* < 0.05). A trend of decrease was seen in LZG and GZG groups comparing with model group (*P* > 0.05), while significant reduction was found in LGZG and LG groups (*P* < 0.05); moreover, LGZG effect was superior to LG group.

### 3.3. Serum Transaminases and Lipid Levels Were Reduced by LGZG

Serum AST in model group was significantly higher than that in normal group (*P* < 0.05), and significant reduction was detected in LGZG, GZG, and LG groups comparing with model group (*P* < 0.05), while no improvement was seen in LZG group (*P* > 0.05). However, serum ALT levels were similar between normal, model, and treating groups.

Serum HDL in model group was significantly lower than that in normal group (*P* < 0.05), while no significant difference was detected for TG. TG levels were significantly reduced in LGZG and LG groups comparing to model group (*P* < 0.05). Serum TC levels in model group were significantly higher than that in normal group (*P* < 0.05), and TC level was significantly reduced in LGZG and LG groups (*P* < 0.05), while no striking decrease was found in LZG and GZG groups (*P* > 0.05, Table 3).

### 3.4. Serum THs Were Increased by LGZG

Serum FT3, T4, and FT4 levels in model group were significantly lower than that in normal group (*P* < 0.05), while only a trend of reduction was found for T3 level (*P* > 0.05). T3, FT3, T4, and FT4 levels were significantly increased in LGZG and LG groups, compared with model group (*P* < 0.05). T4 level was significantly increased in GZG group (*P* < 0.05), but GZG treatment showed no effect on T3, FT3, and FT4 levels (*P* > 0.05). LZG treatment had no effect on all THs (*P* > 0.05, Table 4).

### 3.5. LGZG Improved Fatty Acid Beta-Oxidation via Regulation of TR*β*1 and CPT1A Expression

**Figure 2:**
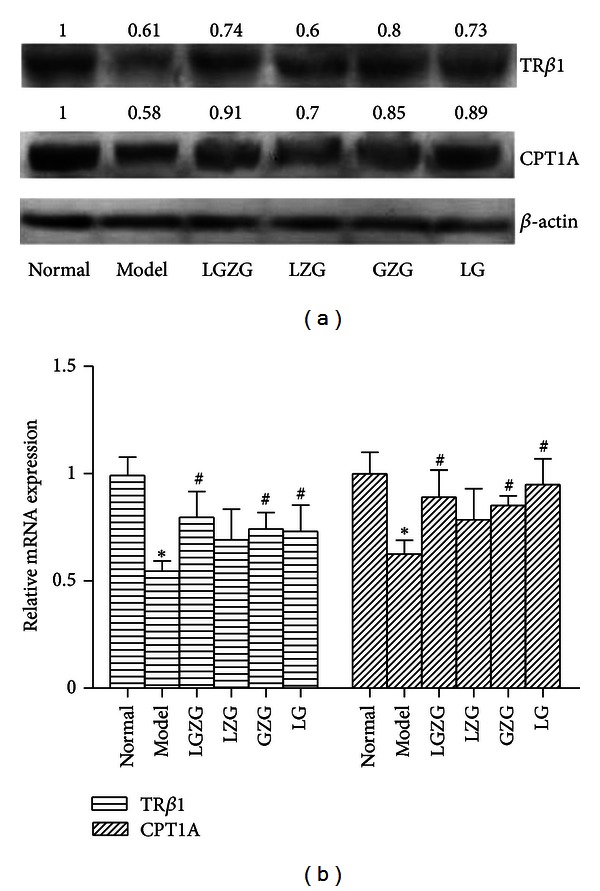
LGZG improved fatty acid beta-oxidation via regulation of TR*β*1 and CPT1A expression. The relative mRNA and protein expression of TR*β*1 and CPT1A were detected by real-time PCR and western blot. (a) The influence of LGZG and its decomposed recipes on the protein expression of hepatic TR*β*1 and CPT1A; (b) the influence of LGZG and its decomposed recipes on the relative mRNA of hepatic TR*β*1 and CPT1A (normalized by *β*-actin). *P < 0.05 versus normal group, ^#^ *P* < 0.05 versus model group.

The gene and protein expression of thyroid hormone receptor *β*1 (TR*β*1) and carnitine palmitoyltransferase-1A (CPT1A) from the liver tissue in Model group were significantly lower than that in normal group (*P* < 0.05) (Figures 2(a) and 2(b)). LGZG, GZG, and LG treatments significantly improved the mRNA and protein expression of TR*β*1 and CPT1A (*P* < 0.05), while no such effect was found in LZG group (*P* > 0.05). LGZG effect on mRNA expression of TR*β*1 was superior to other groups.

### 3.6. LGZG Enhanced Metabolism and Transport of Fatty Acid through Modulation of SREBP-1c, ACSL, and ApoB100 Expression

**Figure 3:**
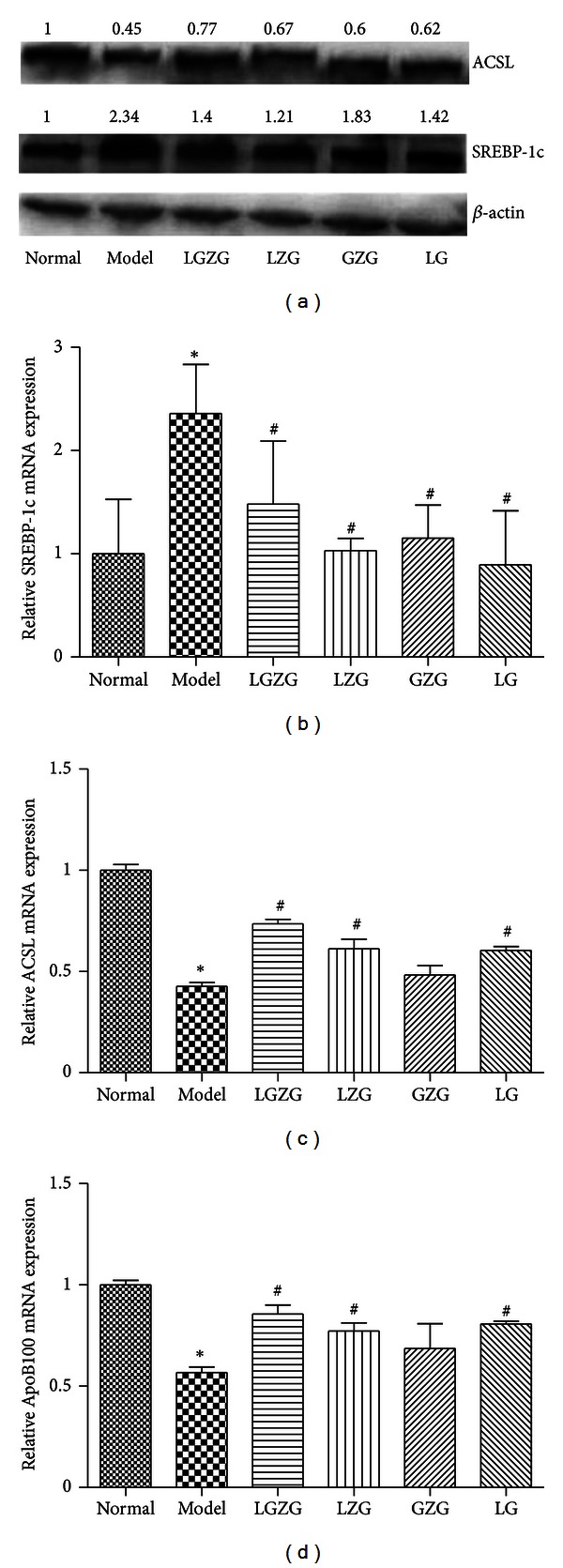
LGZG enhanced metabolism and transport of fatty acid through modulation of SREBP-1c, ACSL and ApoB100 expression. The relative mRNA and protein expression of SREBP-1c, ACSL, and ApoB100 were detected by real-time PCR and western blot. (a) The influence of LGZG and its decomposed recipes on the protein expression of hepatic SREBP-1c and ACSL; (b)–(d) the influence of LGZG and its decomposed recipes on the relative mRNA of hepatic SREBP-1c, ACSL, and ApoB100 (normalized by *β*-actin). *P < 0.05 versus normal group, ^#^ *P* < 0.05 versus model group.

The gene and protein expression of sterol regulatory element-binding protein 1c (SREBP-1c) from the liver tissue in model group were significantly higher than that in normal group (Figures 3(a) and 3(b)),while the treating groups reduced its expression significantly (*P* < 0.05).

Conversely, the gene and protein expression of long-chain acyl-CoA synthetase (ACSL) and apolipoprotein B100 (ApoB100) in model group were significantly lower than that in normal group (*P* <0.05) (Figures 3(a), 3(c), and 3(d)); LGZG, LZG, and LG treatments significantly improved expression of ACSL and ApoB100 (*P* < 0.05), and LGZG was superior to LZG and LG groups; however, GZG treatment showed no such effect (*P* > 0.05).

## 4. Discussion

Lingguizhugan decoction (LGZG) is a classic TCM formula, which has been used to treat obesity and hyperlipidemia in recent years, but the related mechanisms by which LGZG regulates lipid metabolism is yet not clear. In this study, we demonstrated that LGZG significantly attenuated HFD-induced fatty liver disease, as measured by body weight, liver index, EFP/BW, liver injury, and hepatic TG. The possible mechanisms might include increasing serum THs and improving beta-oxidation (via modulation of TR*β*1 and CPT1A expression), metabolism, and transport (through modulation of SREBP-1c, ACSL, and ApoB100 expression) of fatty acid. Our study, for the first time, revealed the mechanisms through which LGZG regulates lipid metabolism; furthermore, we discovered the herbal combination with the properties of warming yang to relieve water retention in the formula and proposed the biological basis of LGZG conventional effect via further study on disassembled formula. It is of great significance in understanding the classic formulas' conventional efficacy.

NAFL is characterized by the accumulation of TGs, which are formed from free fatty acids (FFA) and glycerol within the hepatocyte. There is a positive TG cycle between liver and adipose tissue in physiological conditions. It can only be exported from the liver in very low-density lipoprotein (VLDL) particles after incorporation into the apolipoprotein (ApoB100) because TG is liposoluble and insoluble in body (an aqueous environment). The ability of synthesizing TG in the liver is greater than that of synthesizing ApoB100 and package VLDL then as a result, NAFL commonly occurs in association with imbalance between production of TG and apolipoprotein synthesis. 

FFA and glycerine are hydrolyzed by lipase from TG within the hepatocyte and then released into blood, and FFA generates energy via *β*-oxidation. FFA is firstly translated to activated acyl-CoA before the catabolic oxidation, and the latter one is freely soluble in water. Thus the metabolic activity of FFA is enhanced by catalyzing acyl-CoA synthetase (ACS). 

Our results demonstrated that LGZG, LZG, and LG significantly increased hepatic ACSL (a major isoform of ACS in liver) and ApoB100 in rat models of NAFL. As these three formulas have a common herb *Poria* and the formula without *Poria* (GZG) showed no such effect, we suggested that *Poria* (which is sweet and tasteless in flavor, with a function of drain dampness with bland) is the key factor why a formula relieves water retention. *Poria* might function through promoting package of VLDL from TG, enhancing aqueous solubility and metabolic activity of fatty acid. The major biological basis of relieving water retention is the high expression of ACSL and ApoB100.

LGZG has the function of warming yang to relieve water retention, and there are similarities between TCM yang-warming and THs function. THs show great importance in metabolic regulation as they significantly promote energy metabolism and fat metabolism, and particularly they improve lipid mobilization, reduce fat storage, and accelerate fatty acid oxidation. Liver is an important target organ where THs execute its function by binding to thyroid hormone receptors (TRs) physiologically, and it also plays an important role in the synthesis, transformation, and inactivation of THs. TR*β*1, a major isoform of TRs in liver, is the major modulator in T3 regulation of cholesterol metabolism which plays a key role in hepatic lipid metabolism [14, 15]. Preclinical studies have been conducted for TR*β*1 receptor agonists and showed that they reduced plasma cholesterol and TG. In an animal study, a TR*β*1 receptor agonist (M07811) was found to accelerate mitochondria fatty acid oxidation and alleviate hepatic steatosis [16].

Studies have demonstrated that TR regulated CPT1 expression by binding to TH-response elements (TRE) in the promoter regions of carnitine palmitoyltransferase-1 (CPT1), a rate-limiting enzyme in hepatic mitochondria fatty acid oxidation [17–20]. CPT1 expression is closely related to body fat percentage, and the gene expression is regulated at transcriptional level [21]. High-CPT1 expression is correlated with high decomposition of fatty acid, low body fat percentage, alleviated hepatic steatosis, and delayed occur of fatty liver [22]. Three isoforms of CPT1 are currently known: CPT1A, CPT1B, and CPT1C. CPT1A (liver isoform) is mainly expressed in liver, kidney, and pancreas, with the function of fatty acid *β*-oxidation regulation [23]. The CPT1A expression in NAFLD patients is reduced, and hepatic TG is reduced by increasing CPT1A expression [24]. Hepatic TG level is significantly increased while CPT1A activity is restrained [25]. Enhanced CPT1A activity is correlated with improved fatty acid *β*-oxidation, reduced injury caused by high FFA and TG, and increased TG secretion [26]. Another study has shown strong interaction between CPT1A and ACSL [27]. 

Hashimoto et al. [28] demonstrated that T3 significantly reduced mice SREBP-1c expression via TR*β*1, and this suppression might be caused by TRs and LXR (from SREBP-1c) competition for a DNA binding site [29]. The expression of genes involved in lipid metabolism and glycometabolism is regulated by SREBP-1c, an isoform of sterol regulatory element binding proteins (SREBPs) in liver [30, 31]. Previous study reported that SREBP-1c regulated synthesis and storage of TG in liver [32]. SREBP-1c overexpression can cause dyslipidaemia and lead to lipid accumulation and fatty liver. It was confirmed that hepatic fat content in ob/ob mice with a superimposed knockout of SREBP-1 was significantly lower than that in wide-type controls [33]. SREBP-1c regulates lipid synthesis via transcription regulation of hepatic lipase by changing its mRNA level. Our results demonstrated that LGZG, GZG, and LG significantly increased hepatic TR*β*1 in rat models of NAFLD. As these three formulas have a common herb *Ramulus Cinnamomi* (with a function of warming yang for qi activation), while formula without *Ramulus Cinnamomi *(LZG) showed no such effect and all four formulas showed beneficial effect on CPT1A, we suggested that *Ramulus Cinnamomi* is the key factor why a formula warms yang for qi activation. *Ramulus Cinnamomi* might function through increasing THs, hepatic TR*β*1, and CPT1A expression and enhancing fatty acid *β*-oxidation. Meanwhile, herbal combination (*Rhizoma Atractylodis Macrocephalae* and *Radix Glycyrrhizae*) with the property of fortifying the spleen and replenishing qi might strengthen this effect. The major biological basis to warm yang for qi activation is the high expression of TR*β*1 and CPT1A.

In addition, our results demonstrated that LGZG and LG significantly reduced hepatic TG level in rat models of NAFLD, and formulas without *Poria* (diuresis) or *Ramulus Cinnamomi* (yang-warming for qi activation) showed no such effect. Based on the theory of syndrome differentiation through formula and correspondence of prescription and syndrome, we proposed that water and dampness retention caused by spleen yang deficiency is the basic TCM pathogenesis of NAFLD, and warming yang to relieve water retention is an effective therapeutic principle in NAFLD preventionand treatment. This reflects the therapeutic idea rooted in *Jingui Yaolue*: *conditions with phlegm and fluid retention should be modulated by drugs with warming nature.* The combination of *Poria* and *Ramulus Cinnamomi* might be crucial in LGZG, which is established under the therapeutic principle of Warming Yang to Relieve Water Retention. This formula directly supports the idea that there is a harmonious combination of warming tonification and pathological accumulation elimination. The herbal combination of *Rhizoma Atractylodis Macrocephalae* and *Radix Glycyrrhizae* enhances the effect of warming yang to relieve water retention.

In summary, our study confirmed that LGZG provided significant beneficial effect on HFD-induced rat models of NAFLD, and the mechanisms underlying the effect of LGZG may include increasing THs and improving fatty acid *β*-oxidation and metabolism. LGZG might be an alternative therapy for MS, such as NAFLD, based on the data from the present study. However, further clinical trials about LGZG efficacy and studies about other possible mechanisms are warranted.

## Supplementary Material

An Agilent 1100 HPLC system consisting of a G1354A pump, a G1313A auto-sampler, and a UV Detector was used for all analyses. Chromatographic separations were carried out on an Merck C18 hibar column (4.6 mm×250 mm5 **µ**m) with methanol:acetonitrile:water:acetid acid (15:35:45:0.9,v/v) in the mobile phase at a flow rate of 0.8 mL/min at 25°C for 40 min. The HPLC fingerprint of lingguizhugan decoction revealed the major peaks (glycyrrhizic acid) at 254 nm. The content of glycyrrhizic acid were 0.9548%.

## Figures and Tables

**Table 1:** List of primers.

Gene	Forward primer	Reverse primer	Probe	NCBI RS*
TR*β*1	AATGGGGAAATGGCAGGAC	AAGACATCAGCAGGACGGC	CAGGGCAACCTCCGTGTCATCC	NM_012672.2
CPT1A	ATCACTGGTGTGTTCCCCG	GATCTTTGCGATCATGCCC	ATGGATGAAATCACACCCACCACCA	NM_031559.2
SREBP-1c	GCCATCGACTACATCCGCTT	CAGGTCTTTCAGTGATTTGCTTTT	CAGCACAGCAACCAGAAACTCAAGCA	XM_213329
ApoB100	GCATTCTAACTGCCGAGGG	CAAATGGTTGTGCCGAAAAG	CCATTTAAGTTGGCATTGTGCTCACCA	NM_019287.2
ACSL	ATCTTCCCTGTGGTTCCGAG	TCTGACGATGCCACTGCG	CAAAATCCAACAGCCATCGCTTCAC	NM_012820.1
*β*-actin	AGGGAAATCGTGCGTGAC	CGCTCATTGCCGATAGTG	CTGTGCTATGTTGCCCTAGACTTC	NM_031144.2

*RS: reference sequence.

**Table 2:** The influence of LGZG decoction and its decomposed recipes on rats' body weight, liver index, and EFP/BW (mean ± SD).

Group	*n *	Body weight (gm)	Liver index (%)	EFP/BW (%)
Normal	8	323.25 ± 15.50	3.39 ± 0.11	0.101 ± 0.007
Model	8	341.13 ± 11.99*	4.44 ± 0.09*	0.139 ± 0.008*
LGZG	8	332.13 ± 17.47	4.21 ± 0.20^#^	0.119 ± 0.010^#^
LZG	8	344.75 ± 6.90	4.33 ± 0.11	0.129 ± 0.009
GZG	8	345.75 ± 13.14	4.33 ± 0.11	0.124 ± 0.016
LG	8	348.88 ± 17.23	4.27 ± 0.07^#^	0.121 ± 0.007^#^

*P < 0.05 versus normal group, ^#^ *P *< 0.05 versus model group.

**Table 3:** The influence of LGZG decoction and its decomposed recipes on serum transaminases and lipid levels (mean ± SD).

Group	*n *	ALT (U/L)	AST (U/L)	TG (mmol/L)	TC (mmol/L)	HDL (mmol/L)	LDL (mmol/L)
Normal	8	41.83 ± 4.38	113.28 ± 21.51	1.31 ± 0.43	1.48 ± 0.10	1.39 ± 0.11	0.12 ± 0.02
Model	8	44.18 ± 3.83	128.64 ± 9.91*	1.24 ± 0.30	2.40 ± 0.22*	1.12 ± 0.10*	1.00 ± 0.19
LGZG	8	41.48 ± 3.10	105.41 ± 12.18^#^	0.60 ± 0.16^#^	2.00 ± 0.10^#^	1.03 ± 0.05^#^	0.94 ± 0.12
LZG	8	44.21 ± 3.56	118.10 ± 9.31	0.87 ± 0.13	2.18 ± 0.36	1.08 ± 0.08	1.00 ± 0.27
GZG	8	43.26 ± 4.60	103.90 ± 14.84^#^	0.94 ± 0.23	2.46 ± 0.24	1.09 ± 0.08	1.16 ± 0.22
LG	8	40.90 ± 4.48	105.44 ± 10.25^#^	0.55 ± 0.16^#^	2.04 ± 0.13^#^	1.03 ± 0.04^#^	0.96 ± 0.11

*P < 0.05 versus normal group, ^#^ *P* < 0.05 versus model group.

**Table 4:** The influence of LGZG decoction and its decomposed recipes on serum T3, FT3, T4, and FT4 (mean ± SD).

Group	*n *	T3 (nmol/L)	FT3 (nmol/L)	T4 (nmol/L)	FT4 (nmol/L)
Normal	8	1.02 ± 0.08	4.65 ± 0.61	73.59 ± 5.66	38.44 ± 2.12
Model	8	0.96 ± 0.08	4.06 ± 0.49*	54.97 ± 3.60*	28.38 ± 2.39*
LGZG	8	1.05 ± 0.06^#^	4.56 ± 0.39^#^	66.73 ± 2.58^#^	33.76 ± 1.88^#^
LZG	8	0.97 ± 0.07	4.04 ± 0.43	58.73 ± 4.67	30.31 ± 2.82
GZG	8	1.00 ± 0.10	4.48 ± 0.56	59.35 ± 3.05^#^	29.31 ± 1.66
LG	8	1.10 ± 0.05^#^	5.13 ± 0.30^#^	70.23 ± 4.10^#^	35.33 ± 1.93^#^

*P < 0.05 versus normal group, ^#^ *P* < 0.05 versus model group.

## Abbreviations

ACS: Acyl-CoA synthetase
ACSL: Long-chain acyl-CoA synthetase
ALT: Almandine aminotransferase
ApoB100: Apolipoprotein B100
AST: Aspartate aminotransferase
CPT1: Carnitine palmitoyltransferase-1
CPT1A: Carnitine palmitoyltransferase-1A
EFP/BW: Epididymal fat pad-bodyweight ratio
FT3: Free triiodothyronine
FT4: Free thyroxine
HDL-C: High-density lipoprotein cholesterol
LDL-C: Low-density lipoprotein cholesterol
NAFL: Nonalcoholic fatty liver
SREBP-1c: Sterol regulatory element-binding protein 1c
SREBPs: Sterol regulatory element binding proteins
TC: Total cholesterol
TG: Triglyceride
TRE: TH-response elements
TRs: Thyroid hormone receptors
TR*β*1: Thyroid hormone receptor*β*1
T3: 3,5,3–triiodothyronine
T4: Total thyroxine
VLDL: Very low-density lipoprotein.

## References

[1] Ming-Liang Q, Jing-Yuan M, Jia-Ying W, Xian-Liang W (2011). A Meta-analysis: effect of Linggui Zhugan or added formula on chronic heart failure. *Chinese Journal of Experimental Traditional Medical Formulae*.

[2] Xiao-Yin G, Xiao-Qiu L, Xi-Qing L, He-Wei W, Yan-Jing T (2006). The effect of modification of Lingguizhugan decoction on serum arrial natriuretic factor and cardiac function of congestive heart failure disease of rabbits. *Journal of Emergency in Traditional Chinese Medicine*.

[3] Hai-Yan F, Jin-Ling H, Fang-Fang S (2010). Effects of Linggui Zhugan Decoction on levels of angiotensin II, endothelin-1, tumor necrosis factor-*α* and interleukin-1*β* in rats with chronic heart failure. *Journal of Anhui Traditional Chinese Medical College*.

[4] Ding GA, Yu GH, Liang SC (2006). Jiawei lingguizhugan tang for obesity induced by psychoactive drugs. *Chinese Journal of Clinical Rehabilitation*.

[5] Qi Z, Yong J, Jian-Bin C (2003). The experimental study on the influence of Lingguizhugan Decoction on the hemorheology in hyperlipidemic rats. *Journal of Chengdu Unversity of Tarditional Chinese Medicine*.

[6] Chen DS, Ke B, Huang YJ (2011). Effects of the modified linggui zhugan decoction (see text) combined with short-term very low calorie diets on glycemic control in newly diagnosed type 2 diabetics. *Journal of Traditional Chinese Medicine*.

[7] Wei HF, Liu T, Xing LJ, Zheng PY, Ji G (2009). Distribution pattern of traditional Chinese medicine syndromes in 793 patients with fatty liver disease. *Journal of Chinese Integrative Medicine*.

[8] Miao W, Tao L, Hua-Feng W, Lian-Jun X, Pei-Yong Z, Guang J (2010). Clinical study of “Jiangzhi Granule” and behavioral intervention for nonalcoholic fatty liver disease of phlegm and blood-stasis syndrome. *Shanghai Journal of Traditional Chinese Medicine*.

[9] Liu T, Yang L-L, Zhang L, Song H-Y, Li D-F, Ji G (2012). Comparative study on the effects of different therapeutic methods in preventing and treating nonalcoholic fatty liver in rats. *Journal of Chinese Integrative Medicine*.

[10] Li KG (1995). *The Handouts of Jingui Yaolüe*.

[11] Zong-Hua S, Dong F, Jun-Bo XU, Kai-Shun B (2003). Study on the compatibility and therapeutical basis of composite herbal medicines of Lingguishugan Decoction. *Chinese Traditional Patent Medicine*.

[12] Chen CX (2006). *Pharmacology of Traditional Chinese Medicine*.

[13] Livak KJ, Schmittgen TD (2001). Analysis of relative gene expression data using real-time quantitative PCR and the 2-ΔΔCT method. *Methods*.

[14] Pramfalk C, Pedrelli M, Parini P (2011). Role of thyroid receptor *β* in lipid metabolism. *Biochimica et Biophysica Acta*.

[15] Gullberg H, Rudling M, satló C, Forrest D, Angelin B, Vennstrِm B (2002). Requirement for thyroid hormone receptor beta in T3 regulation of cholesterol metabolism in mice. *Molecular Endocrinology*.

[16] Cable EE, Finn PD, Stebbins JW (2009). Reduction of hepatic steatosis in rats and mice after treatment with a liver-targeted thyroid hormone receptor agonist. *Hepatology*.

[17] Louet JF, Le May C, Pégorier JP, Decaux JF, Girard J (2001). Regulation of liver carnitine palmitoyltransferase I gene expression by hormones and fatty acids. *Biochemical Society Transactions*.

[18] Napal L, Marrero PF, Haro D (2005). An intronic peroxisome proliferator-activated receptor-binding sequence mediates fatty acid induction of the human carnitine palmitoyltransferase 1A. *Journal of Molecular Biology*.

[19] Dann HM, Drackley JK (2005). Carnitine palmitoyltransferase I in liver of periparturient dairy cows: effects of prepartum intake, postpartum induction of ketosis, and periparturient disorders. *Journal of Dairy Science*.

[20] Sim KG, Hammond J, Wilcken B (2002). Strategies for the diagnosis of mitochondrial fatty acid *β*-oxidation disorders. *Clinica Chimica Acta*.

[21] Barger PM, Kelly DP (2000). PPAR signaling in the control of cardiac energy metabolism. *Trends in Cardiovascular Medicine*.

[22] Musso G, Gambino R, Cassader M (2009). Recent insights into hepatic lipid metabolism in non-alcoholic fatty liver disease (NAFLD). *Progress in Lipid Research*.

[23] Drynan L, Quant PA, Zammit VA (1996). Flux control exerted by mitochondrial outer membrane carnitine palmitoyltransferase over *β*-oxidation, ketogenesis and tricarboxylic acid cycle activity in hepatocytes isolated from rats in different metabolic states. *Biochemical Journal*.

[24] Chen J, Xie ZQ, Ye S, Wang K, Wang SD, Zhang QH (2010). Effect of Ginkgo biloba extract (GBE50) on the metabolism of triglyceride in HepG2 cells. *Chinese Pharmacological Bulletin*.

[25] Dobbins RL, Szczepaniak LS, Bentley B, Esser V, Myhill J, Denis McGarry J (2001). Prolonged inhibition of muscle carnitine palmitoyltransferase-1 promotes intramyocellular lipid accumulation and insulin resistance in rats. *Diabetes*.

[26] Stefanovic-Racic M, Perdomo G, Mantell BS, Sipula IJ, Brown NF, O’Doherty RM (2008). A moderate increase in carnitine palmitoyltransferase 1a activity is sufficient to substantially reduce hepatic triglyceride levels. *American Journal of Physiology, Endocrinology and Metabolism*.

[27] Lee K, Kerner J, Hoppel CL (2011). Mitochondrial carnitine palmitoyltransferase 1a (CPT1a) is part of an outer membrane fatty acid transfer complex. *Journal of Biological Chemistry*.

[28] Hashimoto K, Yamada M, Matsumoto S, Monden T, Satoh T, Mori M (2006). Mouse sterol response element binding protein-1c gene expression is negatively regulated by thyroid hormone. *Endocrinology*.

[29] Liu YY, Brent GA (2010). Thyroid hormone crosstalk with nuclear receptor signaling in metabolic regulation. *Trends in Endocrinology and Metabolism*.

[30] Horton JD, Shah NA, Warrington JA (2003). Combined analysis of oligonucleotide microarray data from transgenic and knockout mice identifies direct SREBP target genes. *Proceedings of the National Academy of Sciences of the United States of America*.

[31] Shimano H, Shimomura I, Hammer RE (1997). Elevated levels of SREBP-2 and cholesterol synthesis in livers of mice homozygous for a targeted disruption of the SREBP-1 gene. *Journal of Clinical Investigation*.

[32] Kim JB, Wright HM, Wright M, Spiegelman BM (1998). ADD1/SREBP1 activates PPARgamma through the production of endogenous ligand. *Proceedings of the National Academy of Sciences of the United States of America*.

[33] Yahagi N, Shimano H, Hasty AH (2002). Absence of sterol regulatory element-binding protein-1 (SREBP-1) ameliorates fatty livers but not obesity or insulin resistance in Lepob/Lepob mice. *Journal of Biological Chemistry*.

