# Supplementary material for: Chinese Medicine Formula Lingguizhugan Decoction Improves Beta-Oxidation and Metabolism of Fatty Acid in High-Fat-Diet-Induced Rat Model of Fatty Liver Disease
Source: Evid Based Complement Alternat Med. 2013 May 2;2013:429738. doi: 10.1155/2013/429738 (PMC3664975; doi:10.1155/2013/429738)
Supplement: Supplementary file 1 — An Agilent 1100 HPLC system consisting of a G1354A pump, a G1313A auto-sampler, and a UV Detector was used for all analyses. Chromatographic separations were carried out on an Merck C18 hibar column (4.6 mm×250 mm5 µm) with methanol:acetonitrile:water:acetid acid (15:35:45:0.9,v/v) in the mobile phase at a flow rate of 0.8 mL/min at 25°C for 40 min. The HPLC fingerprint of lingguizhugan decoction revealed the major peaks (glycyrrhizic acid) at 254 nm. The content of glycyrrhizic acid were 0.9548%. [file 429738.f1.pdf]

# Supplement 1

## Quality control of Lingguizhugan Decoction under High Performance Liquid Chromatography (HPLC)

Standardization of extract by HPLC glycyrrhizic acid was purchased from Shanghai R&D Centre for standardization of Chinese Medicines (Shanghai, China), and the purity was higher than 98.0%. HPLC-grade reagents were purchased from Burdick & Jackson. An Agilent 1100 HPLC system consisting of a G1354A pump, a G1313A auto-sampler, and a UV/VIS Photodiode Array G1315B Detector was used for all analyses. Chromatographic separations were carried out on an Merck C18 hibar column (4.6 mm×250mm，5μm) with methanol:acetonitrile : water :acetid acid(as solvent ) (15:35:45:0.9,v/v) in the mobile phase at a flow rate of 0.8 mL/min at 25℃ for 40 min. Twenty µL (after a high speed centrifugation) of the sample was injected, and the signals were detected at 254 nm with UV detection. The HPLC fingerprint of lingguizhugan decoction revealed the major peaks (glycyrrhizic acid) at 254 nm. The content of glycyrrhizic acid were 0.9548%.

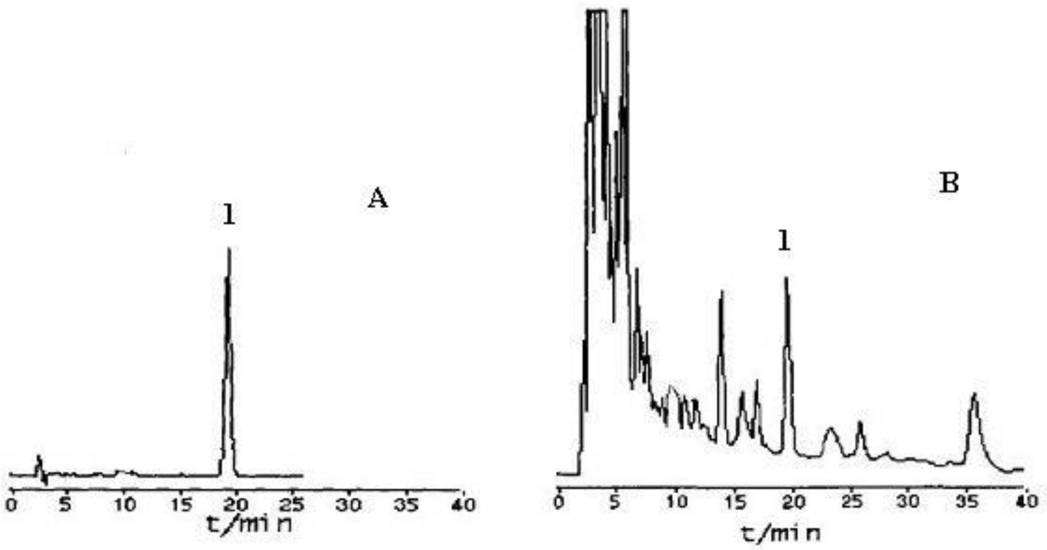


Figure 1 HPLC chromatograms of Lingguizhugan Decoction

A.Reference substance;B. Lingguizhugan Decoction 1. glycyrrhizic acid
